# Effect of Footwear Modifications on Oscillations at the Achilles Tendon during Running on a Treadmill and Over Ground: A Cross-Sectional Study

**DOI:** 10.1371/journal.pone.0152435

**Published:** 2016-03-24

**Authors:** Ilka Meinert, Niklas Brown, Wilfried Alt

**Affiliations:** Institute of Sport and Exercise Science, University of Stuttgart, Stuttgart, Germany; University of Rome Foro Italico, ITALY

## Abstract

**Background:**

Achilles tendon injuries are known to commonly occur in runners. During running repeated impacts are transferred in axial direction along the lower leg, therefore possibly affecting the oscillation behavior of the Achilles tendon. The purpose of the present study was to explore the effects of different footwear modifications and different ground conditions (over ground versus treadmill) on oscillations at the Achilles tendon.

**Methods:**

Oscillations were measured in 20 male runners using two tri-axial accelerometers. Participants ran in three different shoe types on a treadmill and over ground. Data analysis was limited to stance phase and performed in time and frequency space. Statistical comparison was conducted between oscillations in vertical and horizontal direction, between running shoes and between ground conditions (treadmill versus over ground running).

**Results:**

Differences in the oscillation behavior could be detected between measurement directions with peak accelerations in the vertical being lower than those in the horizontal direction, *p* < 0.01. Peak accelerations occurred earlier at the distal accelerometer than at the proximal one, *p* < 0.01. Average normalized power differed between running shoes (*p* < 0.01) with harder damping material resulting in higher power values. Little to no power attenuation was found between the two accelerometers. Oscillation behavior of the Achilles tendon is not influenced by ground condition.

**Conclusion:**

Differences in shoe configurations may lead to variations in running technique and impact forces and therefore result in alterations of the vibration behavior at the Achilles tendon. The absence of power attenuation may have been caused by either a short distance between the two accelerometers or high stiffness of the tendon. High stiffness of the tendon will lead to complete transmission of the signal along the Achilles tendon and therefore no attenuation occurs.

## Introduction

During running, impact forces occur at every step with the foot hitting the ground. These forces are then transferred along the lower leg in longitudinal direction and cause oscillations of soft tissues surrounding bony structures, including muscles and tendons [1]. The magnitude of impact forces can be modified by changes in running speed [2] and through the use of different running shoes [3]. Shock absorbing materials are used in running shoes, aiming to limit these forces [4]. Differences in loading rate and impact magnitude also occur when running over ground compared to running on a treadmill [5,6]. Therefore every running shoe may lead to specific input characteristics to the system when running at different running speeds and on different grounds.

Due to its location at the distal end of the lower leg, the Achilles tendon is strongly affected by impact forces. Loading of this tendon can reach up to 12.5 times body weight during running [7,8] and elite male distance runners have a 52% risk of Achilles tendinopathy [9]. According to Lohrer (2006), overuse injuries of the Achilles tendon are the most common reason for dropouts in athletic careers, especially in track and field [10]. Impact forces and loading rates are often suggested to affect injury rates at the lower extremity [11,12]. The influence of these forces and the resulting oscillations of soft tissue compartments were recently studied [13–15]. However, their effects on sports-related injuries seem to be poorly understood. In the past, high impact forces were thought to cause harm to the musculoskeletal system [16,17]. Newer findings suggest positive effects of undamped impacts, serving as input signals to the system [18]. In mechanical components, resonant oscillations are expected to destabilize a system, possibly resulting in catastrophic damage. In biological systems, however, the mechanical properties of soft tissues (viscoelastic component), including the Achilles tendon, may be altered through neuromuscular adaptations [19,20]. Even though specific resonance frequencies exist for tendons, they can be altered through changes in joint angles and muscular contractions [21]. Kinematics as well as muscular activity of the lower extremity differ substantially when running on a treadmill compared to running over ground [22,23]. These differences in running technique may also lead to alterations in the vibration behavior at the Achilles tendon.

The input signal to the Achilles tendon results from ground reaction forces which may be altered through changes in running shoes. Shoe modifications lead to changes in the input frequency during walking [24]. Also, changes in transmissibility were found during drop jumps when wearing different shoes [25]. Therefore, the purpose of this study was to explore the effects of different running shoes and different surface conditions (treadmill versus over ground) on oscillations at the Achilles tendon during running. We expected alterations in cushioning and stabilizing components of the shoe as well as alterations of ground conditions to result in changes of oscillations at the Achilles tendon.

## Materials and Methods

20 male rear foot runners were included in the present study (age: 22.7 ± 2.9 years, height: 1.83 ± 0.06 m, weight: 79.9 ± 7.51 kg, running distance per week: 20.35 ± 8.11 km, running experience: 5.4 ± 3.2 years). Subjects were recruited from the university’s student population and stated to be free of any neurologic or orthopedic complaints at the lower extremity. The principles declared in the Declaration of Helsinki were followed when conducting this study. In agreement with the Professional Code for Physicians in Germany (§15 (1)), no patients or children were participating in this study, the integrity of the study participants was not intervened and no body materials were extracted. Therefore, the study was not required to be evaluated by an ethics committee. Prior to measurements each subject was informed about the procedures and signed an informed consent. Each participant was assigned a number and data collection and data handling were performed in de-identified form.

Subjects were given a six minute warm up period at a self-selected running speed to get accustomed to the treadmill (Woodway ERGO XELG 90®, Woodway USA Inc., Waukesha, WI, USA). Past research has shown a successful familiarization to treadmill running after six minutes [26]. Subjects were asked to run at a running speed of 2.9 m/s on the treadmill and over ground. A predefined speed was chosen as a dependency of the evaluated data on running speed was found during previous pilot measurements. All subjects perceived 2.9 m/s as a comfortable running speed, which was manageable for all endurance levels. During the trials, study participants wore two differently configured running shoes as well as one neutral all-purpose shoe as a reference (NS; Adidas Gazelle®, Adidas, Herzogenaurach, Germany). The order in which the shoes were worn as well as the ground conditions were randomized. NS is characterized by no specific arch support of the foot bed, no medial wedges and very hard damping material in the shoe’s sole. A modular running shoe (Runaissance 3.0®, Newline, Vodskov, Denmark) was used to provide two different running shoe configurations. This system allows the modification of three components of the shoe: foot bed, medial wedges and cushioning. We used the modular system to provide two different configurations of this shoe: one with high arch support, medial wedges (4 mm), heel wedges (2cm) and soft damping material (Con1), the other with low arch support, no medial wedges and hard damping material (Con2). All shoes were tested according to ASTM F-1976 standards. The results of these tests are shown in Table 1.

**Table 1:** Damping characteristics of shoe conditions used in the present study.

SHOE	MAXIMUM FORCE [N]	G-SCORE (PEAK)	PEAK-TO-PEAK RATIO [%]
**CON1**	867.3	14.3	55.6
**CON2**	914.9	15.6	55.5
**NS**	1546.5	24.1	50.7

**Fig 1:**
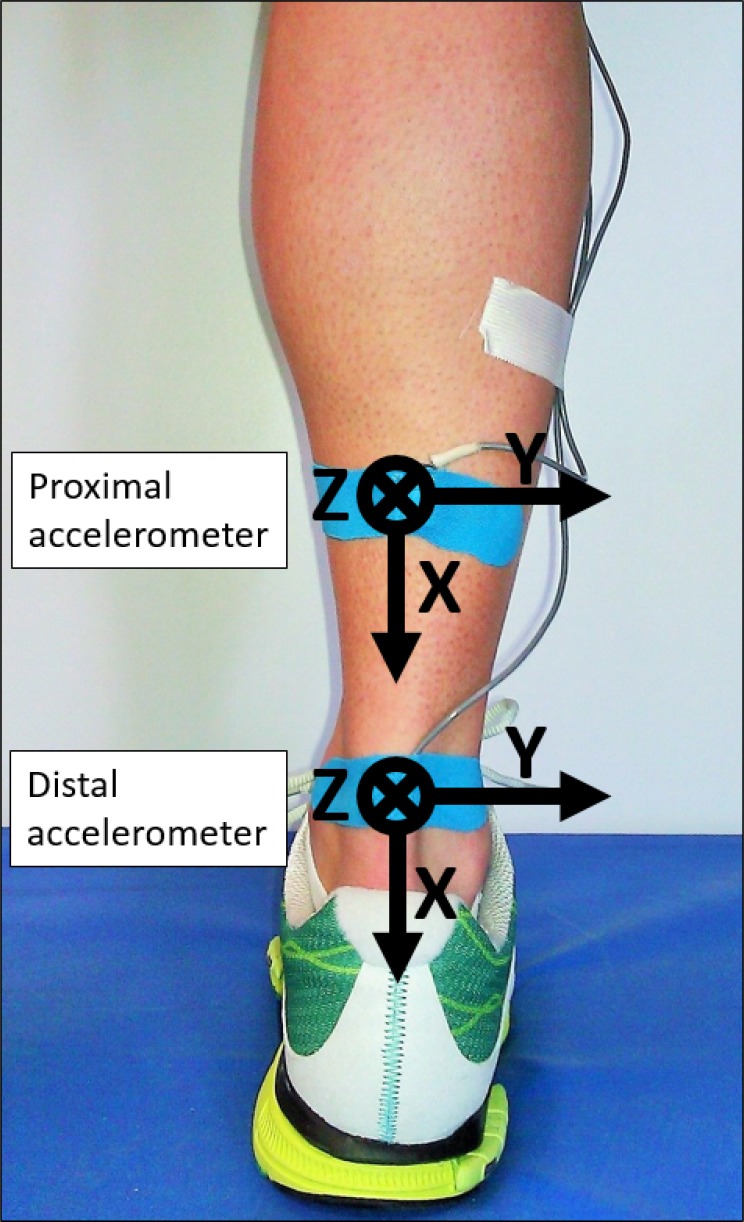
Placement of accelerometers. Both sensors were attached to the skin overlaying the Achilles tendon. The proximal accelerometer was located at 36% of tibial length while the distal accelerometer was affixed at 26% of tibial length.

Subjects were given a 1 minute familiarization period while running in each shoe condition, followed by a 10 second measurement period. Over ground running was performed on a 30 meter runway on concrete floor. Concrete floor is a ground condition comparable to asphalt roads, on which most long distance runners perform their training. Therefore, locomotion on concrete floor very closely resembles road running. Trials were accepted if the time needed to cover this distance was between 9 sec (3.3 m/s) and 11 sec (2.7 m/s). Step detection took place using an in shoe plantar pressure system (F-Scan®, Tekscan Inc., South Boston, MA, USA). Data included in further analysis was limited to stance phases of the right foot. Two tri-axial accelerometers (Noraxon Corporate, Scottsdale, AZ, USA) were attached to the skin overlaying the right Achilles tendon using double sided tape and overstretched using kinesio tape. Kinesio tape was needed to ensure firm attachment of the sensors to the skin and was chosen as it allows diffusion of moisture (sweat) and is known to have similar characteristics as skin. It can therefore be assumed that oscillations at the Achilles tendon were not affected by the tape. Due to their small size (2.03 cm x 1.52 cm x 0.76 cm) and mass (2.8 g) the sensors are easy to attach to the skin and are able to measure accelerations up to 6 G. Achilles tendon length correlates with tibia length [27]. In order to assure comparable accelerometer locations between subjects, tibial length was determined in a sitting position from ground to Caput fibulae. The distal accelerometer was then attached at 26% of the tibial length and the proximal accelerometer at 36% (determined from the ground up). The positions of the two accelerometers was tested in pilot measurements and ensured the collection of data, which was not influenced either by the nearby heel cap of the shoe or by the cuff of the plantar pressure measurement system. The devices were aligned with each other and parallel to the Achilles tendon. Sensor application is depicted in Fig 1. Acceleration data at the Achilles tendon were sampled with a frequency of 1500 Hz using Noraxon Telemyo 2400 G2 (Noraxon Corporate, Scottsdale, AZ, USA) while pressure data were synchronously sampled at 100 Hz.

Data of each accelerometer were analyzed individually and separately in the vertical x-direction as well as in the yz-plane (resulting vector). Absolute peak accelerations and the time to peak acceleration (ttpeak) following first foot contact (ffc) were determined as dependent variables. Oscillation frequencies are partially generated by motions of body segments and are therefore present at all structures, muscles as well as other tissue like the Achilles tendon. To limit the effect of these oscillations, data were high pass filtered with a cut off frequency of 10 Hz as frequencies below 10 Hz typically contain movement artifacts [13]. Spectral analysis of the acceleration data was performed according to Boyer & Nigg (2006) [14]. This study was chosen as a reference because a similar experimental setup was used with subjects performing a heel-toe run and because data analysis included both, fundamental frequency results and the quantification of oscillation attenuation. In short, the data were zero-padded and transformed to the frequency domain using Fast Fourier Transformation (Matlab R2013b, Mathworks, Natick, MA, USA). Average signal power was calculated for each accelerometer and in each measurement direction (vertical x -direction and horizontal yz-plane resultant). Power was then normalized to stance phase and normalized by peak power from both accelerometers of each trial. Normalized power will further be denoted as *P(f)* and was analyzed in a low-frequency interval (10–25 Hz), medium-frequency interval (25–50 Hz), high-frequency interval (50–100 Hz) and a highest-frequency interval (> 100 Hz). The dominant frequency in each trial was determined as the highest peak in each power spectrum and was used as dependent variable.

Transfer functions resemble mathematical relations between input and output signals of a dynamic system in the frequency domain and are a frequently used engineering term in systems theory. In the present study the transfer function *H(f)* resembles the ratio between normalized power of proximal and distal accelerometer signals. It quantifies the power attenuation between the two accelerometers.

$$H(f)=10log\left[\frac{{P(f)}_{proximal}}{{P(f)}_{distal}}\right]$$ (1)

Repeated measures ANOVAs were performed to analyze differences in the dependent variables between running shoes, ground conditions, accelerometer locations, and frequency intervals. Separate ANOVAs were calculated for peak acceleration, ttpeak, dominant frequency and average normalized power as a comparison between these variables would have been meaningless. The assumption of normality was checked and the data were found to meet the criteria. Violations of sphericity were controlled using Maulchy’s test of sphericity and either Greenhouse-Geisser or Huynh-Feldt corrections were applied according to Girden (1992) [28]. If a Greenhouse-Geisser epsilon of > 0.75 was found, the Huynth-Feldt corrected value was used for that parameter. Otherwise the Greenhouse-Geisser corrected value was used. Post hoc tests were performed using modified t-tests with Bonferroni correction. All statistical calculations were completed using SPSS (SPSS 21, IBM, Armonk, NY, USA) and the alpha-level was set at 0.05.

## Results

### Time domain

**Fig 2:**
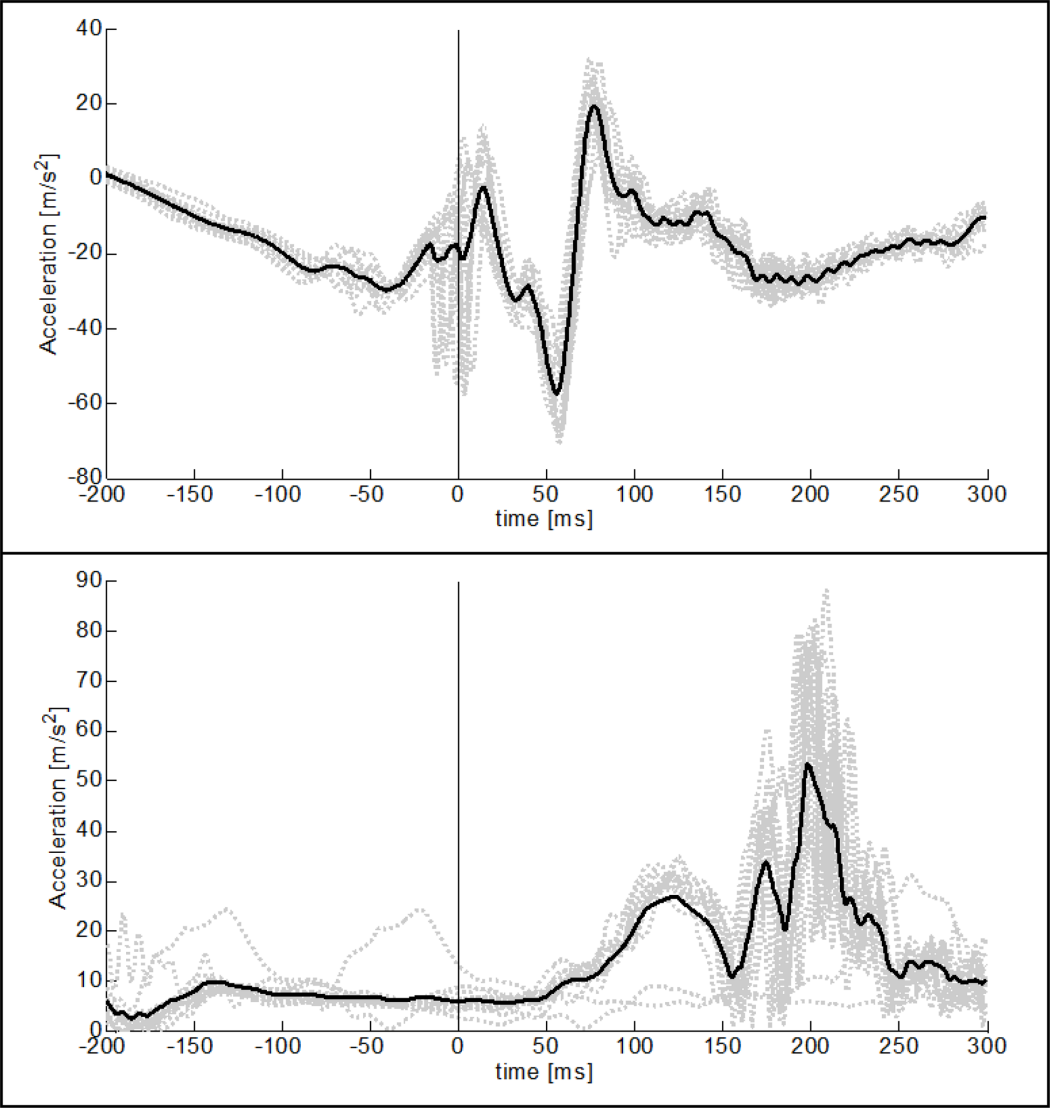
Characteristic acceleration signals. Acceleration signals recorded at the Achilles tendon while running on a treadmill shown for one representative subject. Grey lines show the data obtained from each step while black lines depict mean curves, averaged over all steps. ffc occurred at time = 0 ms.

The acceleration signal of one representative subject obtained with the distal accelerometer in the vertical x-direction as well as the resulting acceleration in the horizontal yz-plane is shown in Fig 2. The acceleration data shown in the graphs were recorded with the subject running on a treadmill. A time frame of 200 ms before and 300 ms after ffc is depicted.

**Fig 3:**
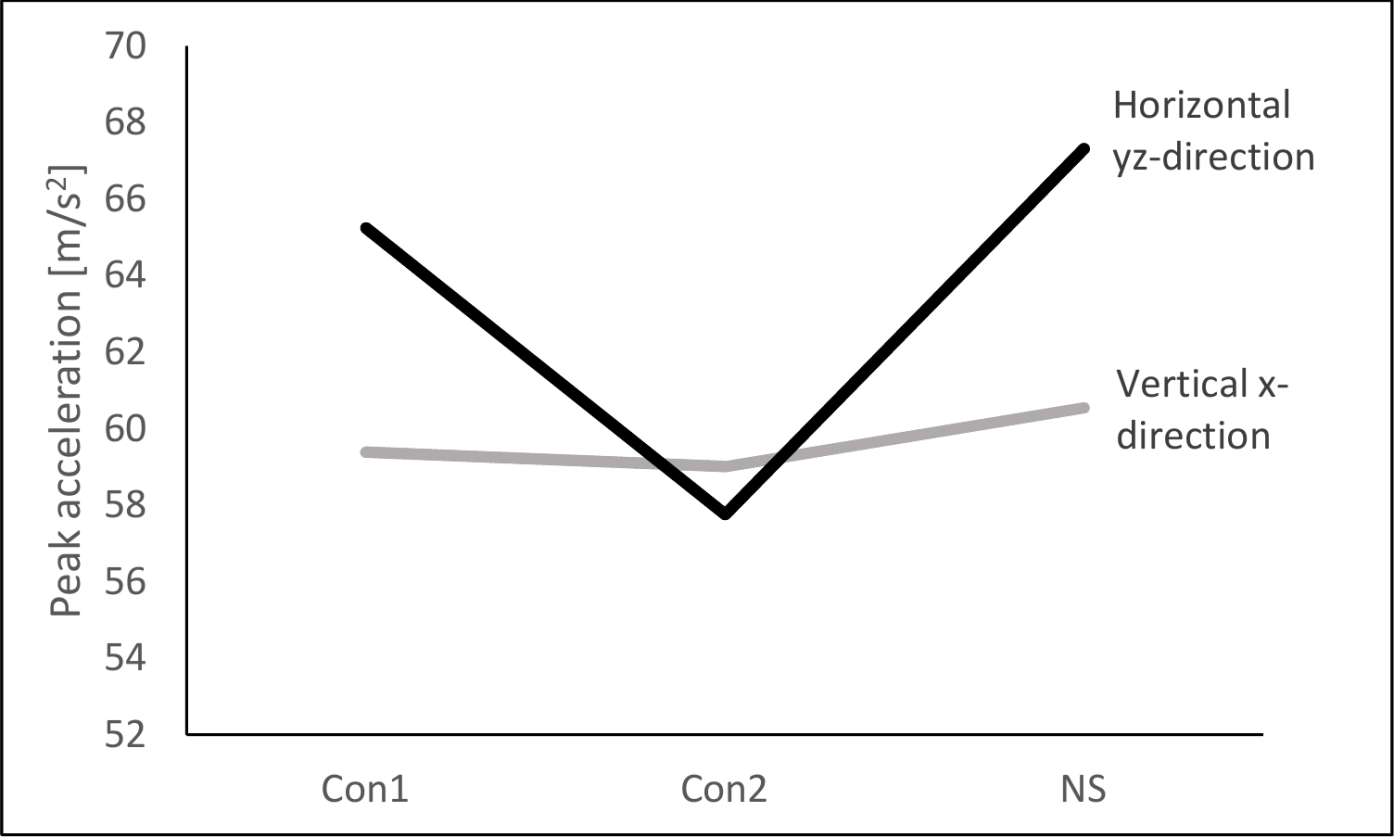
Interaction effect of Direction*Configuration. Peak accelerations measured at the Achilles in horizontal and vertical direction. Measurements were performed while running in three different types of footwear.

Direction was found to have a significant main effect on peak accelerations, F(1, 19) = 6.62, *p* = 0.02, η_p_^2^ = 0.26. Peak accelerations in the vertical x-direction (59.64 ± 12.47 m/s^2^) were significantly lower than those in horizontal yz-plane (63.44 ± 16.65 m/s^2^), *p* = 0.02. A significant interaction effect was detected for Direction*Configuration, F(2, 38) = 8.27, *p* = 0.01, η_p_^2^ = 0.30. Peak accelerations in vertical x-direction were barely influenced by the shoe condition while those in horizontal yz-plane were decreased when running in Con2 (57.75 ± 18.59 m/s^2^) compared to Con1 (65.24 ± 13.49 m/s^2^) or NS (67.32 ± 17.86 m/s^2^) as shown in Fig 3.

**Fig 4:**
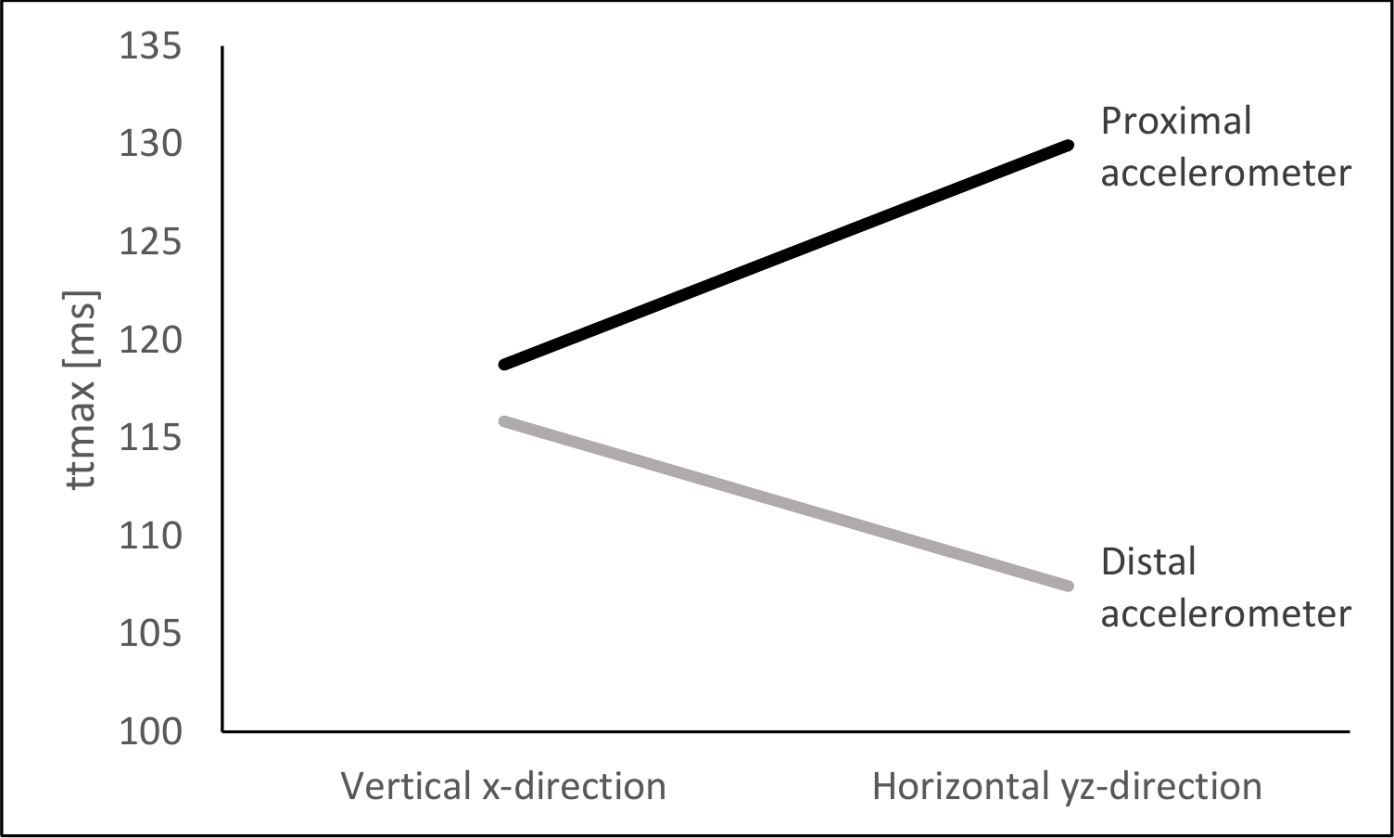
Interaction effect of Location*Direction. ttpeak at different accelerometer locations (proximal and distal) and in different measurement directions.

Accelerometer location was found to have a significant main effect on ttpeak, F(1, 19) = 8.00, *p* = 0.1, η_p_^2^ = 0.30. ttpeak was shorter at the distal (111 ± 74 ms) compared to the proximal accelerometer (124 ± 74 ms), *p* = 0.1. Therefore, peak accelerations occurred earlier at the distal accelerometer than at the proximal sensor. A significant interaction effect could be proven for Location*Direction, F(1, 19) = 16.60, *p* < 0.01, η_p_^2^ = 0.47. ttpeak in vertical x-direction was comparable between the two accelerometers (distal: 115 ± 78 ms; proximal: 118 ± 73 ms). However, for the proximal accelerometer a profound elongation of ttpeak was found in the horizontal yz-plane (129 ± 76 ms) while a reduction of ttpeak occurred at the distal accelerometer (107 ± 70 ms, Fig 4).

### Frequency domain

**Fig 5:**
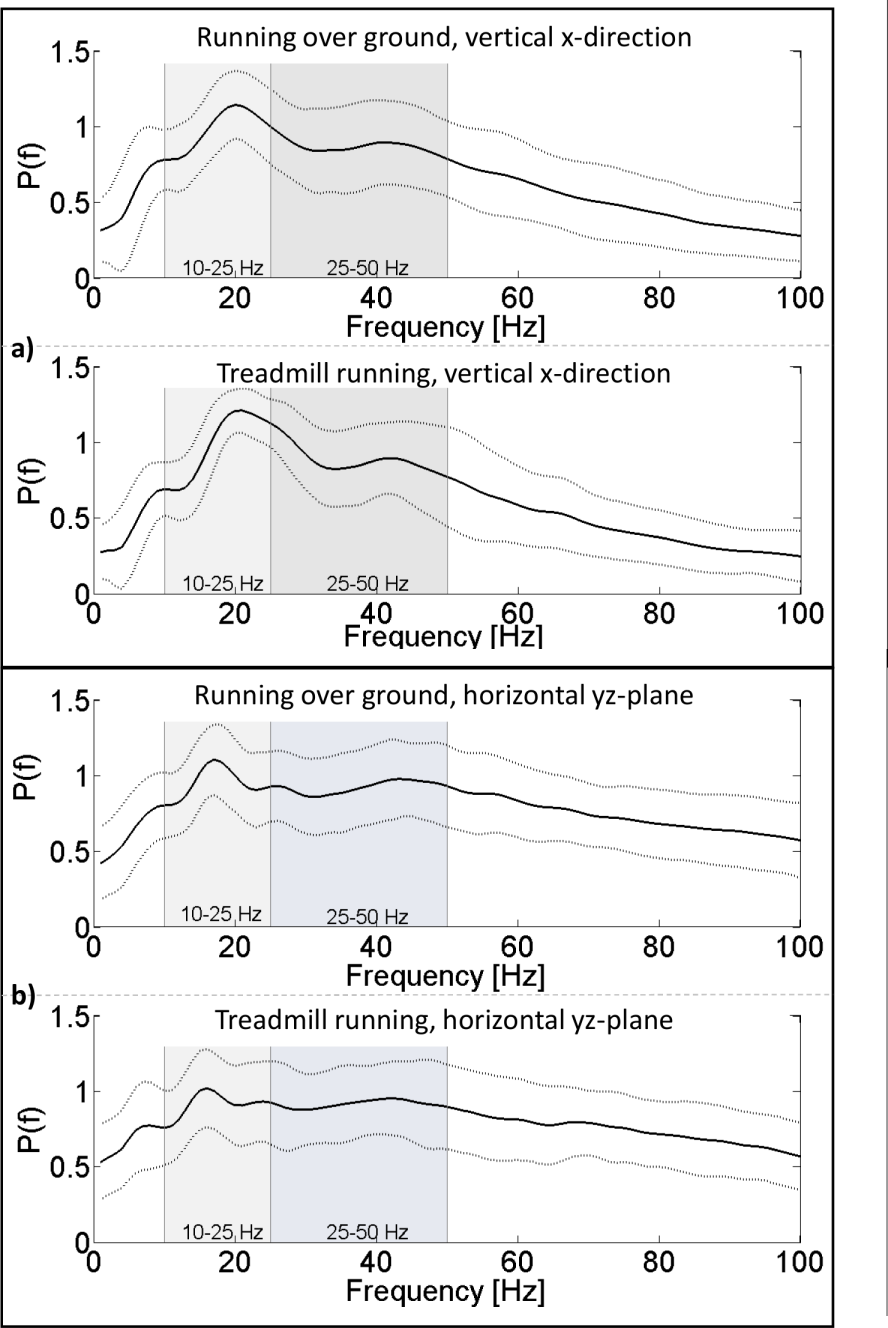
Normalized power spectrum of the distal accelerometer. Average power spectrum for running over ground and running on a treadmill, separated in two measurement directions. Graph a) shows data in the vertical x-direction while graph b) shows data in the horizontal yz-plane. Solid lines indicate mean values of all study participants, dashed lines indicate SDs. Low-frequency and medium-frequency intervals are depicted in the graphs.

**Fig 6:**
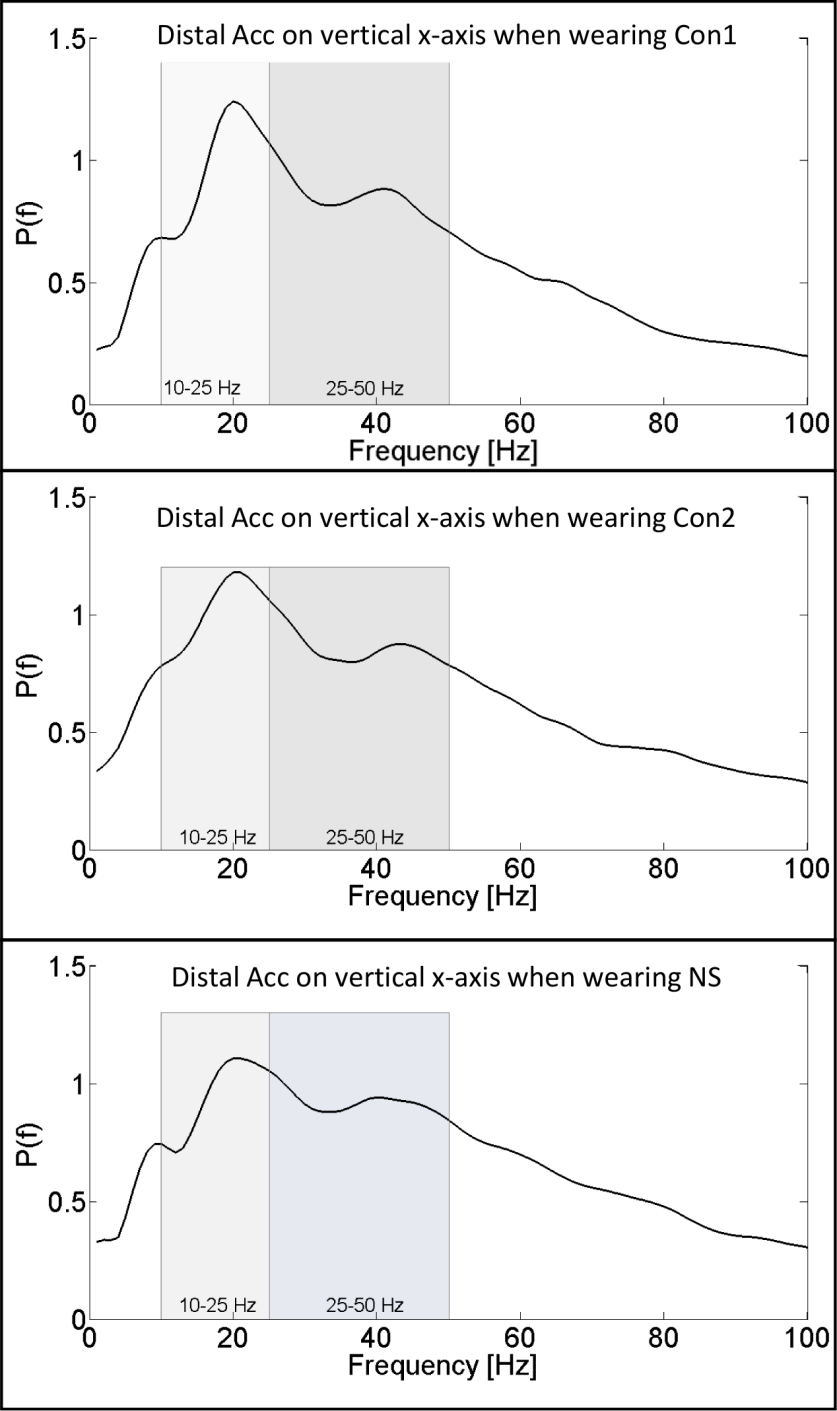
Normalized power spectrum of the distal acceleration signal in vertical x-direction. Power is averaged over ground conditions (treadmill and over ground) and separately shown for three different running shoe configurations. Low-frequency and medium-frequency intervals are depicted in the graphs.

Mean normalized power spectra of the acceleration signals obtained while running at two different ground conditions (over ground and treadmill) are shown in Fig 5. Each signal is plotted in the horizontal yz-plane and the vertical x-direction. While data in the horizontal yz-plane show a more even power distribution with a moderate peak in the medium-frequency interval, the power spectra obtained from data in the vertical x-direction show two characteristic peaks, one in the middle-frequency interval and the other in the high-frequency interval. When comparing the power spectra obtained during treadmill running to those obtained during over ground running, no distinct differences in their characteristics can be found. Fig 6 shows mean normalized power spectra of the distal accelerometer in vertical x-direction while wearing different shoe configurations.

The average dominant frequency of oscillations at the Achilles tendon was found to be 30.8 ± 17.9 Hz. A difference in the dominant frequency was only found between measurement directions, F(1, 19) = 20.89, *p* < 0.01, η_p_^2^ = 0.52, not between oscillations induced by running at different grounds, in different shoes or between the two accelerometer locations. The dominant frequency of oscillations in vertical x-direction was 25.6 ± 11.7 while it was 34.6 ± 20.3 in the horizontal yz-plane. Significant main effects on normalized power were found for configuration, F(2, 38) = 25.82, *p* < 0.01, η_p_^2^ = 0.58, ground, F(1, 19) = 7.30, *p* = 0.01, η_p_^2^ = 0.28, frequency intervals, F(1.99, 42.13) = 977.63, *p* < 0.01, η_p_^2^ = 0.98, and accelerometer location, F(1, 19) = 34.54, *p* < 0.01, η_p_^2^ = 0.97.

**Fig 7:**
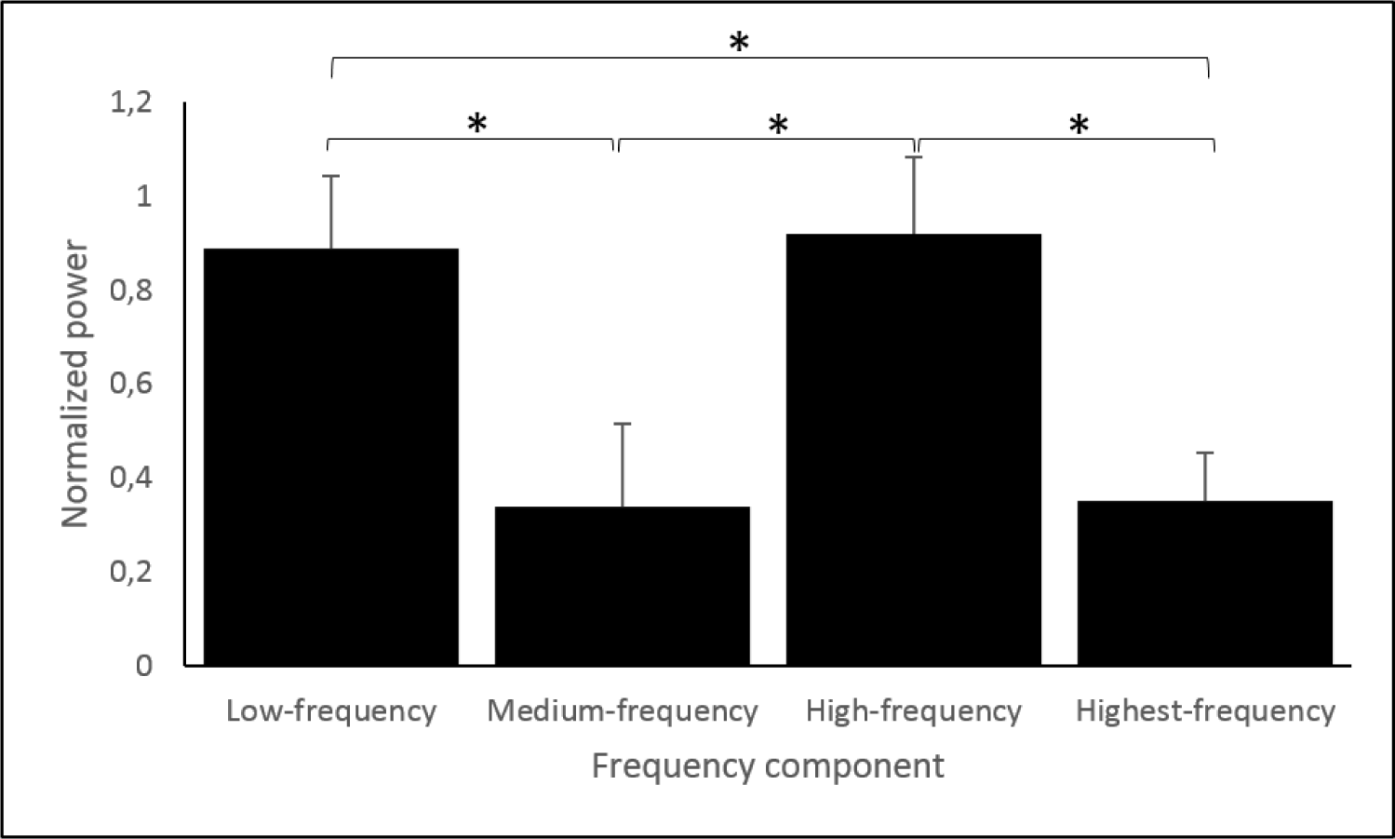
Average normalized power in different frequency intervals. Power was averaged over all subjects and all trials, both accelerometers are included. Bars represent mean values while error bars show SDs. Stars indicate significant differences.

Average normalized power was significantly higher when running in NS (0.7 ± 0.2) compared to Con1 (0.6 ± 0.1; *p* < 0.01) and when running in Con2 (0.6 ± 0.2) compared to running in Con1 (*p* < 0.01). A significant mean difference of 0.02 (95%CI [0.04:0.01]) in normalized power was found between running trials performed on a treadmill (*p* = 0.01) and those performed over ground with signal power being slightly lower when running over ground. Normalized power was highest in the high-frequency interval (0.9 ± 0.2) and lowest in the medium-frequency interval (0.3 ± 0.2). Fig 7 illustrates the differences between frequency intervals, all significant differences are indicated by *p*-values < 0.01. Average normalized power was 0.3 (95%CI [0.25:0.29]) lower in the signal of the distal accelerometer (0.5 ± 0.1) compared to the signal of the proximal accelerometer (0.8 ± 0.2).

**Fig 8:**
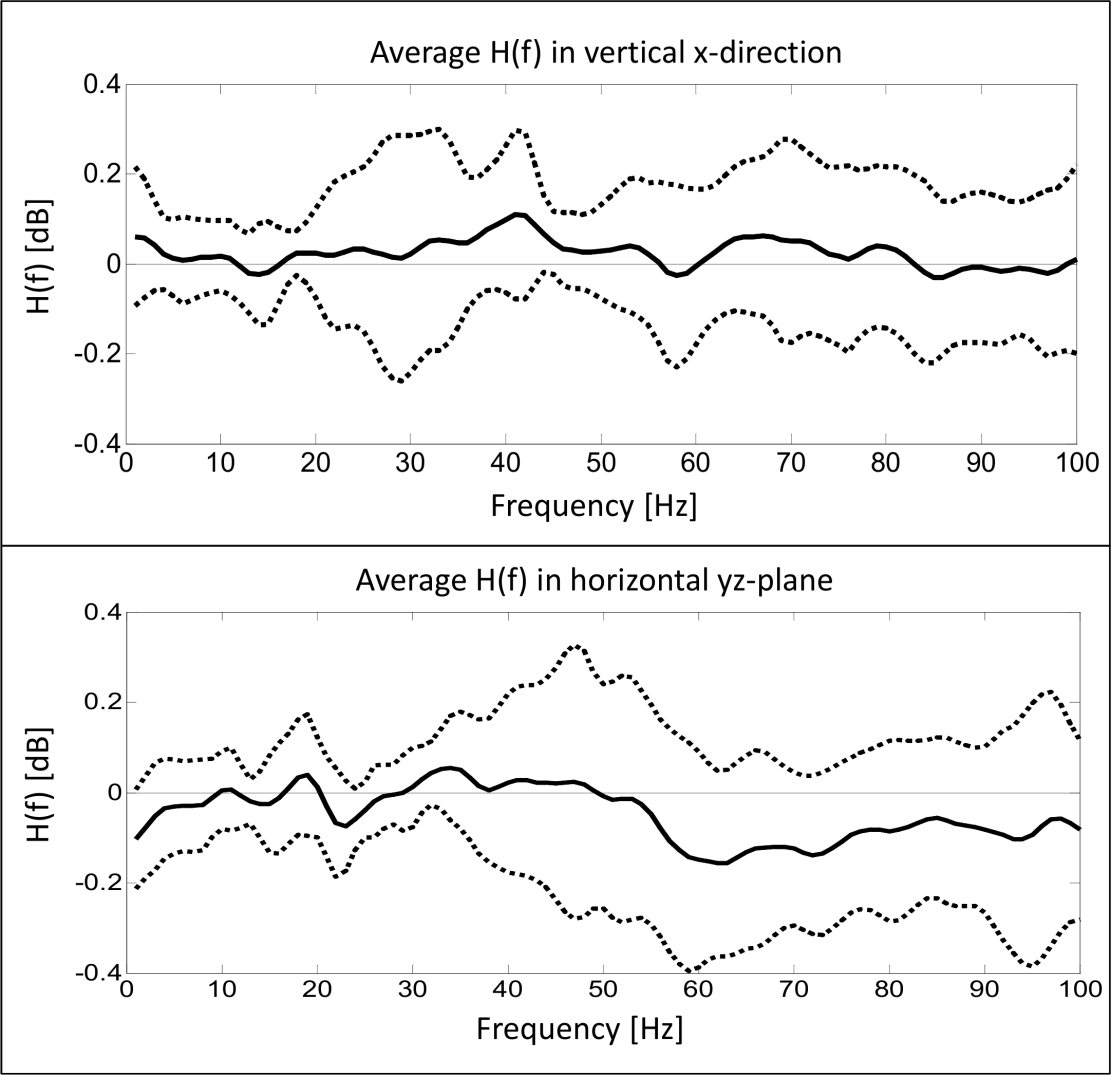
Average transfer function. Power attenuation between distal and proximal accelerometer data. Solid lines show mean data in both measurement directions while dashed lines represent SDs.

In Fig 8 the mean transfer function in both measurement directions is shown in logarithmical plotting for better clarity. In both measurement directions only minimal power attenuation can be seen in the graphs. While barely any damping characteristics can be observed in the vertical x-direction, the power of the proximal accelerometer is very slightly attenuated in the proximal accelerometer data in the horizontal yz-plane in frequency components above 50 Hz.

## Discussion

The purpose of the present study was to test the effects of different footwear modifications and different ground conditions on oscillations at the Achilles tendon. In contrast to our expectations, ground conditions (treadmill versus over ground) did not have an effect on oscillations in time space and produced only slight variations in frequency space. Even though a significant modification of signal power could be detected depending on the ground condition, a change of 0.02 seems extremely small. It will therefore most likely not be of biomechanical relevance. We originally expected differences in ground conditions to lead to variations in running technique and therefore to alterations in the vibration behavior at the Achilles tendon. However, movement pattern fixation is known to occur in experienced runners, especially in running style parameters [29]. Therefore, the experienced runners included in the present study may have held on to their running style, independent of ground condition. Results on Achilles tendon oscillations obtained from treadmill experiments may likely be transferred to over ground running.

The power spectra of the present study show a surprisingly large amount of power in the frequency band of 0–10 Hz, while a high pass filter with a cut off frequency of 10 Hz was applied. This filter was used to minimized accelerations that are due to motions of the lower leg during running, rather than accelerations due to vibrations at the Achilles tendon. An ideal filter would have resulted in zero power at frequencies ≤ 10 Hz. In reality, however, filters do not cut off data abruptly. A frequency attenuation falls off rather slowly with increasing frequency, which can be inspected by the signal dropping off beneath the cut off frequency, as desired [30,31].

While differences between the data obtained from the two accelerometers were found in the time domain, no differences could be proven in the frequency domain. In contrast to the results of Boyer & Nigg (2006) we did not detect an attenuation of power between distal and proximal accelerometer locations [14]. Therefore, the signal power of the two accelerometers did not differ. This effect may have been influenced by the relatively close locations of the two accelerometers in the present study. Step detection was performed using an in-shoe pressure system, which included the mounting of a cuff on the lower leg. This cuff restricted the accelerometer locations at the Achilles tendon. Also, tendon length was estimated by measuring tibial length, which may not be a highly accurate appraisal. The use of an ultrasound system could provide a more accurate definition of the Achilles tendon length and therefore allow a more precise placement of the accelerometers [32].

The stiffness of the underlying material may have influenced the detected attenuation. Boyer & Nigg (2006) studied oscillations at the M. Quadriceps femoris while we collected data at the Achilles tendon [14]. Stiffness of muscle tissue highly depends on its activation level while tendons are known as a relatively stiff material [33,34]. They may therefore transfer the input signal without significant attenuation. Future research should investigate the power attenuation at a farther distance between two accelerometers at the Achilles tendon. Increasing the distance between the two accelerometers may also lead to larger differences in ttpeak of the two sensors. In the present study ttpeak was significantly shorter at the distal accelerometer, which is in agreement with the expected impact transmission along the lower leg following heel contact. It therefore confirms the assumption of impact transmission in longitudinal direction of the Achilles tendon.

Overall, peak normalized power occurred in the medium-frequency interval (25–50 Hz) while average normalized power was lowest in this interval. The normalized power spectra do not show different characteristics when comparing over ground running to treadmill running (Fig 5). As mentioned above, a minor decrease in signal power was detected when running over ground. Ground reaction forces are known to differ when running on a treadmill compared to over ground running, therefore leading to altered input to the system [35,36]. These differences in the system’s input signal may have been encountered with specific muscle activity to ensure a stable oscillation behavior at the Achilles tendon, independent of ground condition.

Oscillations in the vertical x-direction caused a more distinct peak in the medium-frequency interval compared to those in the horizontal yz-plane. The input, possibly characterized by more pronounced first impacts of the foot to the ground, may have led to differences between the power spectra properties of the two measurement directions. In the horizontal measurement direction, a resulting acceleration vector was used for calculations while in the vertical measurement direction, a single vector was used. This may have led to changes in the signal amplitude but not in its frequency. Although the power spectrum obtained in the horizontal yz-plane is more evenly spread over a larger variety of frequencies compared to that in vertical x-direction, the maximum peaks can be found in the medium-frequency interval for both measurement directions. It should be noted that past research has either focused on the vertical direction when analyzing soft tissue oscillations or did not specify measurement directions, although accelerometers with multiple dimensions were used [14,20,25]. Comparisons with values from previous studies should therefore be made with caution.

Variations in shoe configurations led to changes in average normalized power. Overall, signal power decreased with increased damping of the shoe. Due to the modification of both, shock absorption and foot position (wedges), it is impossible to determine a single component that caused the changes in average power. Medial wedges and arch support may have influenced the foot’s position leading to variations in the point of load application during stance. However, if a runner remains in his common movement pattern, these support elements may have caused an increase in ROM of the foot, from a more pronounced supination during early stance to the same pronation values as without wedges. An increased ROM might dampen the input signal and therefore lead to decreased power at the Achilles tendon. Heel wedges were utilized for Con1, which was found to lead to decreased signal power at the Achilles tendon. These wedges represent a common component in the treatment of Achilles tendon complaints, aiming at decreased tension at the tendon. If the strain at the tendon is lower, oscillation amplitudes may increase while frequencies decrease. Heel wedges may not influence amplitude and frequency at the same amount, therefore resulting in lower average power at the Achilles tendon. Fu et al. (2013) studied amplitude and frequency of ground reaction forces during drop jumps with subjects wearing basketball shoes or a minimally-cushioned reference shoe [25]. Harder damping material led to increases in peak vertical ground reaction forces, peak loading rate and ground reaction force frequencies. Lower amplitude and frequency of the input signal will likely lead to lower amplitude and frequency of oscillations at the Achilles tendon. In the present study, this effect was proven for subjects wearing Con1, resulting in lower average signal power. In summary, the Achilles tendon was possibly under different tension, experienced different impact frequencies and was positioned differently. It can therefore only be determined that the combination of those modifications affected the power at the tendon. A single source causing the difference cannot be specified with the present setup. Nor can the possibility that only the combined modifications leads to a change be eliminated.

No effect of shoe modifications on resonance frequency was proven in the present study. Our findings are in agreement with those of Fu et al. (2013) who did not find differences in the resonance frequency when wearing a basketball or a control shoe during drop jumps [25]. Anticipatory muscle activity of the runners may have prevented an effect of shoe modifications on resonance frequency as well as power attenuation. Subjects participating in the present study were experienced runners. Therefore precise motor control during this well-known movement can be assumed, possibly damping oscillations at the Achilles tendon more effectively than differences in the damping characteristics of a running shoe.

It should be noted that subjects were given a familiarization period in each running shoe before the measurements were conducted. This may have led to an optimized muscle tuning while running in each footwear. Our results may have been different if no familiarization period was given, leading to an unexpected change in shoe conditions which could have led to less effective neuromuscular control. A lack of muscle adaptation to the unexpected shoe modifications may lead to changed impact forces, resulting in increased soft tissue oscillations and increased oscillations at the Achilles tendon [37].

## Conclusion

First insight was gained in the oscillation behavior occurring at the Achilles tendon during running. In conclusion, oscillations were found to vary between measurement directions, sensor locations and if different footwear was worn. Only very slight variations could be detected if running over ground compared to treadmill running. While peak accelerations reach the distal end of the Achilles tendon faster compared to the proximal end, no clear attenuation of oscillations could be proven along the tendon. It can therefore be summarized, that the Achilles tendon is homogeneously affected by the magnitude and frequency of oscillations but varies in the timing of these vibrations.

### Limitations

Even though skin movement artifacts are known to be a common source of error when analyzing structures which lay underneath the skin, it is currently unknown how much influence the relative movement between the Achilles tendon and the skin had on the accelerations measured in the present study. Further research is needed in order to clarify the extent to which accelerations obtained through skin-mounted accelerometers correspond to accelerations of the Achilles tendon itself. In real life situations, runners will perform this activity for much longer durations. Prolonged running will lead to fatigue, which is known to decrease sensorimotor control. Therefore, the vibration behavior at the Achilles tendon may be affected more strongly with longer durations of a run, causing increased fatigue and leading to less effective neuromuscular control to tune vibrations. It also remains unknown whether our findings can be transferred to less experienced runners or subjects with a less precise motor control.
